# Combination of curaxin and tyrosine kinase inhibitors display enhanced killing of primitive Chronic Myeloid Leukaemia cells

**DOI:** 10.1371/journal.pone.0266298

**Published:** 2022-03-31

**Authors:** Stella Pearson, Anthony D. Whetton, Andrew Pierce

**Affiliations:** Stem Cell and Leukaemia Proteomics Laboratory, The University of Manchester, Withington, Manchester, United Kingdom; Universita degli Studi di Firenze, ITALY

## Abstract

Despite the big increase in precision medicine targeted therapies developing curative treatments for many cancers is still a major challenge due mainly to the development of drug resistance in cancer stem cells. The cancer stem cells are constantly evolving to survive and targeted drug treatment often increases the selective pressure on these cells from which the disease develops. Chronic myeloid leukaemia is a paradigm of cancer stem cell research. Targeted therapies to the causative oncogene, BCR/ABL, have been developed but drug resistance remains a problem. The introduction of tyrosine kinase inhibitors targeting BCR/ABL were transformative in the management of CML. However, patients are rarely cured as the tyrosine kinase inhibitors fail to eradicate the leukaemic stem cell which often leads to loss of response to therapy as drug resistance develops and progression to more fatal forms of acute leukaemia occurs. New treatment strategies targeting other entities within the leukemic stem cell either alone or in combination with tyrosine kinase are therefore required. Drawing on our previous published work on the development of potential novel targets in CML and other myeloproliferative diseases along with analysis of the facilitates chromatin transcription (FACT) complex in CML we hypothesised that curaxin, a drug that targets the FACT complex and is in clinical trial for the treatment of other cancers, could be of use in the treatment of CML. We therefore assessed the curaxin CBL0137 as a new agent to extinguish CML primitive cells and show its ability to preferentially target CML cells compared to healthy control cells, especially in combination with clinically relevant tyrosine kinase inhibitors.

## Introduction

The oncogene BCR/ABL is the hallmark of Chronic Myeloid Leukaemia (CML) (reviewed in Soverini et al [1]). The introduction of tyrosine kinase inhibitors (TKIs) targeting this constitutively activated protein has been transformative in the treatment of CML. Whilst these drugs induce haematological, cytogenetic and molecular remissions in the majority of patients, they fail to cure the disease due to the continued presence and persistence of leukemic stem cells (LSC) from which the disease develops [2–4]. TKI stopping trials initiated in France (STIM) [5] and subsequently in Australia (TWISTER) [6] and the UK (Destiny) [7] report relapse rates of around 50% within 12 months following drug discontinuation. The presence of quiescent CML LSCs [8,9] which are not reliant on BCR/ABL activity for survival [2,10] has been proposed as the reason why CML is resistant to TKIs [11]. This persistence of LSC in the bone marrow may eventually result in either *BCR*/*ABL*-related or -unrelated mutational events leading to drug resistance and/or a differentiation blockade (manifesting as blast crisis). The Failure of TKIs to eliminate the LSCs, plus the development of drug resistance and transformation to a more fatal disease make CML an exemplar of cancer stem cell research highlighting the difficulties encountered in precision medicine as applied to cancer treatment. It has been argued that CML cannot be cured by TKIs alone but what is needed are new drugs exploiting differences between CML and normal haematopoietic stem cells [12].

Curaxins are being used successfully in other leukemias and solid tumours to combat drug resistance (eg Clinical Trial Identifier: NCT01905228 and NCT02931110). Curaxins target the facilitates chromatin transcription (FACT) complex and are reported to activate p53, inhibit NF-kβ and be involved in MYC driven transcription [13,14]. Quinacrine, from which curaxins were derived, inhibits β-catenin [15] and it is known that the β-catenin pathway is both essential in CML and critical in TKI resistance [16]. Since β-catenin is redundant in the adult haemopoietic system it has been proposed that a combination of β-catenin inhibitors and TKIs may be useful to eradicate the LSC [16]. We have previously reported that simultaneously targeting both p53 and MYC, two reported targets of curaxin, preferentially extinguishes the leukaemic stem cell in CML [17]. Further we have published work demonstrating the potential of curaxin treatment in another myeloproliferative syndrome Juvenile Myelomonocytic leukaemia [18]. As such we hypothesised that curaxin may be of use in the treatment of CML. We therefore investigated the levels of FACT proteins and the reported targets of curaxin in CML before assessing the ability of the curaxin CBL0137 to extinguish CML primitive cells. We demonstrate the ability of CBL0137 to target CML versus cells from healthy controls, especially in combination with TKIs.

## Materials and methods

### Patient material

Written informed consent was obtained for all samples and all research was in compliance with the ethical and legal framework of the Human Tissue Act. Experiments had ethical approval from the NRES committee of the regional NHS health research authority (14/LO/0489 and 17/LO/0888). Primary CML samples were obtained from the Manchester Cancer Research Centre Biobank (HTA 30004) following authorisation by the Tissue Biobank’s scientific sub-committee. Control samples were surplus cells isolated from Leucocyte cones from healthy individuals undergoing leukapheresis during cell donation within the NHS Blood and Transplant Service. The CD34^+^ cell population were enriched using CliniMACS (Miltenyi Biotec) according to standard protocols and previously described [19].

### Protein measurements

Protein expression was assessed using flow cytometry with the Novocyte flow cytometer (ACEA Biosciences) using FloJo software or western blot analysis using standard protocols as previously described [20]. Cellular fractionation was undertaken with a kit from Active Motif (Rixensart) with modifications as previously described [21]. Antibody details are shown in Table 1.

**Table 1:** Antibodies used in the study.

Antigen	Assay	Company and Catalogue number	Research Resource Identifiers	Dilution
Actin-HRP	WB	Sigma A3854	AB_262011	1/50000
β catenin	WB	Cell Signalling #8480	AB_11127855	1/750
β catenin	WB	Proteintech 51067-2-AP	AB_2086128	1/5000
CD235a - Pacific Blue	FC	Biolegend #349107	AB_11219199	1/100
CD31 –FITC	FC	Biolegend #303119	AB_10643590	1/20
CD33—PE-Cy7	FC	Biolegend #366617	AB_2566419	1/20
CD34 –PE	FC	Biolegend #343605	AB_1732033	1/20
HSP90	WB	Enzo AD1-PA-835	Not available	1/1000
IκB-α	WB	Santa Cruz sc-371	AB_2235952	1/100
LC3B	WB	Sigma L7543	AB_796155	1/200
Myc	FC, WB	Cell Signalling #5605	AB_1903938	1/200–1/500
NF-ĸβ p65	FC, WB	Santa Cruz sc-372	AB_632037	1/200–1/750
NF-ĸβ p65 phospho Ser 536	FC	Santa Cruz sc-33020	AB_2179018	1/200
NF-ĸβ1 p105/p50	WB	Cell Signalling #13586	AB_2665516	1/100–1/1000
NF-ĸβ2 p100/p52	WB	Cell Signalling #4882	AB_10695537	1/100–1/750
Non-phospho Ser 37/41 β catenin	WB	Abcam #246504	Not available	1/1000
Non-phospho Ser 45 β catenin	WB	Cell Signalling #19807	AB_2650576	1/500
Phospho-Histone H2A.X	WB	Cell Signalling #9718	AB_2118009	1/750–1/1000
SSRP1	FC, WB	Cell Signalling #13421	AB_2714160	1/100–1/1000
SUPT16H	FC, WB	BioLegend # 607001	AB_315688	1/100–1/1000
Anti Mouse IgG, HRP linked	WB	GE Healthcare NA931	AB_772210	1/5000
Anti Rabbit IgG, HRP linked	WB	GE Healthcare NA934	AB_772206	1/5000

Table detailing the assays (FC = Flow cytometry, WB = western blot), suppliers, Research Resource Identifiers and dilutions of the antibodies used in the study.

### Cell based assays

K562 cell proliferation was measured using WST-1 reagent (Roche Diagnostics). 100μl of K562 cells at 2 x10^4^/ml were plated in triplicate into 96 well cell culture plates and drug or carrier control added. Following incubation for 24 or 48 hours at 37°C in 5% CO_2_ 10μl of WST-1 reagent was added. After 3 hours incubation with the WST-1 reagent absorbance was measured at 450nm on a 800TS microplate reader (BioTek). Colony forming assays on primary patient samples were performed in methylcellulose complete media (R&D systems) supplemented with 2u/ml EPO with CD34^+^ cells at a density of 3000cells/ml as previously described [22]. To assess retention of self-renewal capacity the resulting colonies at day 7 were replated in methylcellulose in the absence of any drugs and colonies counted at 14 days. Annexin V apoptosis and cell surface marker assays were performed in liquid culture. CD34+ cells were cultured in IMDM, 20% fetal calf serum, rhIL-3 (20ng/ml), rhGM-CSF (10ng/ml), rhSCF (50ng/ml) and Epo (2u/ml) for 72hrs. Cells were stained with cell surface marker antibodies (Table 1) prior to staining with Annexin V (Invitrogen #17-8007-74). Hoechst 33258 (Thermofisher H3569) was added at 2ug/ml prior to measuring fluorescence on an LSR II (Becton Dickinson). Results were analysed using FloJo software.

## Results and discussion

The two components of the FACT complex (SSRP1 and SUPT16H) are up-regulated at the mRNA level in quiescent CML stem/progenitor cells [23] (S1 Fig). Given these data and our observations on the therapeutic advantage of targeting p53 and MYC in the treatment of CML [17] and polycythemia vera [19] along with targeting the FACT complex in JMML [18] we hypothesised that targeting the FACT complex with curaxin would be of benefit in CML.

As correlation between mRNA and protein levels in oncogene expressing cells is limited [24,25] we compared the expression of the two FACT components (SSRP1, SUPT16H), and MYC and NF-ĸβ at the protein level in CD34+ cells isolated from patients with CML and healthy controls. Details of CML patient material are given in Table 2. Control samples were cells isolated from leucocyte cones from healthy individuals undergoing leukapheresis within the NHS Blood and Transplant Service.

**Table 2:** Patient data.

Sex	Age	Diagnosis	Treatment at time of sampling	Experiment
M	71	CML	Haematological response to Imatinib but no cytogenetic or molecular response switched to Dasatinib then Nilotinib	CF
M	21	CML	Diagnostic sample	CF, FC, AV
M	16	CML	Diagnostic sample	CF, FC, AV
F	61	CML	Diagnostic sample	FC, AV
M	17	CML	Rasburicase and hydroxyurea	CF, FC, AV
F	16	CML	Dasatinib	CF
M	20	CML	Diagnostic sample	CF, FC, AV

Relevant Patient information is shown and which samples were used for each experiment. CF—Colony forming assays, AV–Annexin V assays, FC—Flow cytometric assessment of protein expression.

**Fig 1:**
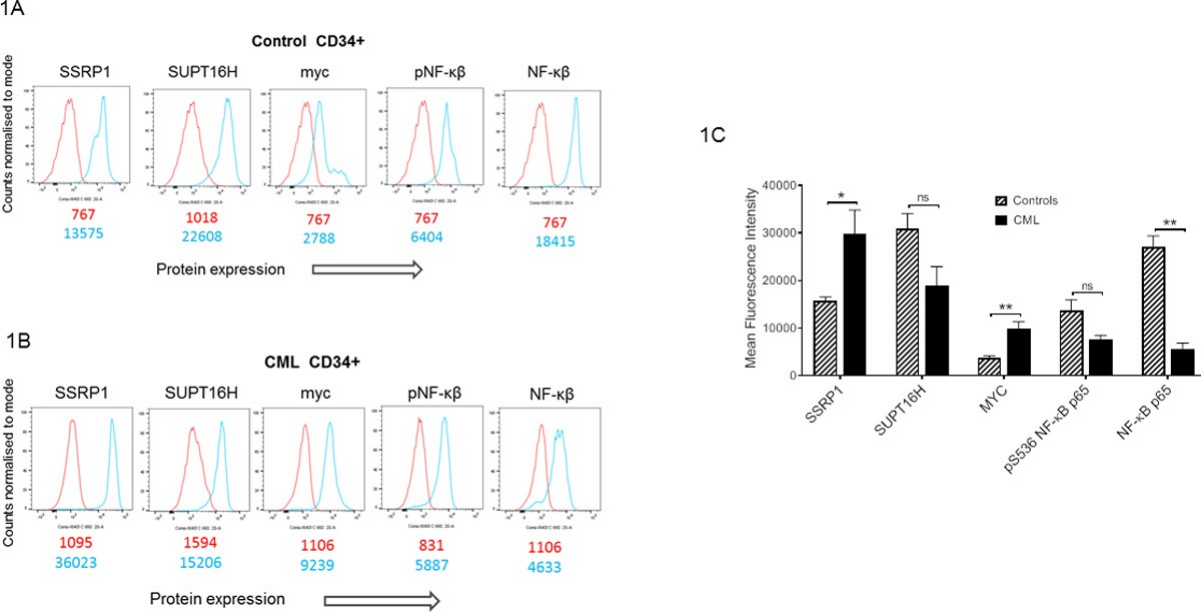
The expression of targets of CBL0137 are perturbed in patients with CML. CD34^+^ cells were enriched from control (Fig 1A) and CML patients (Fig 1B) using CliniMACS (Miltenyi Biotec) and stained with the antibodies to the proteins shown and expression levels analysed on a Novocyte flow cytometer (ACEA Biosciences) using FloJo software. Representative FACS plots are shown (Fig 1A and 1B) with staining with secondary antibody alone illustrated in blue and the staining achieved with the primary antibody shown in red. Amalgamated data (Fig 1C) is displayed as median fluorescent intensity±SEM (n = 4–5 for healthy controls, n = 3 for CML).

In concordance with Affer et al [23], who observed an increase in SSRP1 mRNA expression in quiescent CML stem cells (S1 Fig), we observed an increase in SSRP1 protein expression in CD34+ cells isolated from patients with CML when compared to CD34+ cells from healthy controls (Fig 1A–1C). Furthermore, we demonstrated increased levels of MYC and reduced NF-ĸβ protein expression in CD34+ cells isolated from patients with CML (Fig 1A–1C).

**Fig 2:**
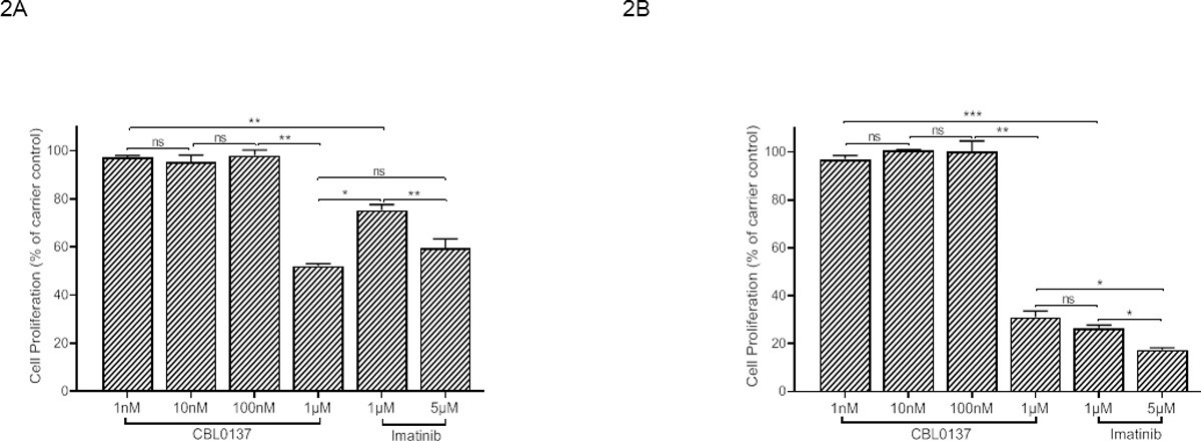
CBL0137 inhibits proliferation of BCR/ABL expressing cells. 2x10^3^ K562 cells were seeded in triplicate in 100μl in a 96 well plate and the effect of a range of concentrations of CBL0137 or 1μM and 5μM Imatinib on the proliferation assessed at 24 (Fig 2A) and 48 hours (Fig 2B) using a WST-1 assay (Roche Diagnostics). Results are expressed as a percentage absorbance of the carrier control and are the mean+/-SEM n = 3. The results of a t-test are shown; ns not significant, *< 0.05, **<0.01, ***<0.001.

To identify an effective dose range for the curaxin CBL0137 in BCR/ABL expressing cells we undertook proliferation assays utilising the cell line K562 in the presence of CBL0137 and compared its inhibitory ability to the frontline CML drug Imatinib. Following 24 hours (Fig 2A) treatment 1μM CBL0137 displayed an equivalent inhibition of proliferation as 5μM Imatinib whilst at 48 hours (Fig 2B) 5μM Imatinib displayed a slightly greater level of inhibition with 1μM CBL0137 being equivalent to 1μM Imatinib.

**Fig 3:**
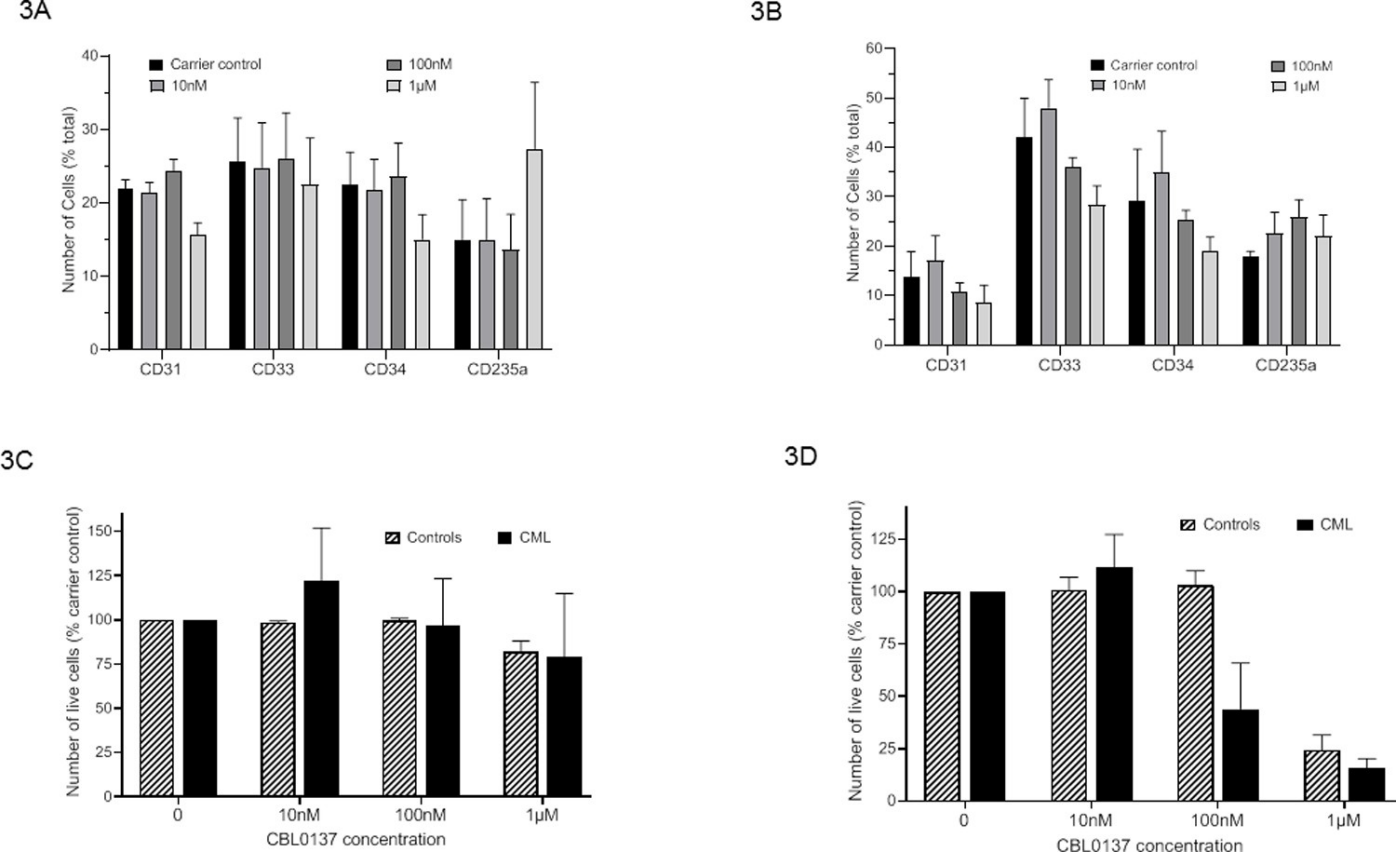
CBL0137 has distinct effects upon different myeloid cell populations in cells from healthy controls and CML patients. CD34+ cells isolated from CML patients and healthy controls were cultured in IMDM, 20%(v/v) fetal calf serum, rhIL-3 (20ng/ml), rhGM-CSF (10ng/ml), rhSCF (50ng/ml) and Epo (2u/ml) for 72hrs in the presence of 0, 10nM, 100nM or 1μM CBL0137. Cells were stained with cell surface marker antibodies shown prior to staining with Annexin V and Hoechst 33258. Fluorescence was measured on an LSR II (Becton Dickinson) and results analysed using FloJo software. The results are displayed as the number of cells in each myeloid cell population as a percentage of the total cell population for healthy controls (Fig 3A) and CML (Fig 3B) and as the number of live cells (as a percentage of the carrier control) in the whole population (Fig 3C) or the CD34+ population (Fig 3D). Results shown are mean±SEM n = 3 for CML and n = 4 for controls.

Having established that the targets of curaxin are perturbed at the protein expression level in CML (Fig 1C) and identified an appropriate dose range for CBL0137 (Fig 2A and 2B) we treated CD34+ cells isolated from CML patients and healthy controls in liquid culture with a reduced range of doses of CBL0137 to investigate whether it had differential effects in terms of inducing apoptosis or driving differentiation in defined myeloid cell subpopulations. The distribution of the different myeloid cell populations obtained from CD34+ cells isolated from healthy controls (Fig 3A) and CML patients (Fig 3B) culture for 72 hours in the presence of varying doses of CBL0137 are shown (Fig 3). No significant differences were seen suggesting CBL0137 does not drive differentiation. Considering the cells in totality CBL0137 does not have a significant effect on the level of apoptosis (Fig 3C) however there is a trend visible of increased apoptosis in the higher doses of CBL0137 in the CD34+ cell population (Fig 3D). Further there is a marked difference in the level of apoptosis between CD34+ cells isolated from CML patients and healthy controls at 100nM CBL0137. Significant changes in cell viability were only observed at 1μM CBL0137 in the healthy controls (Fig 3D). 100nM CBL0137 therefore appears to offer a therapeutic window and induces cell death via apoptosis whilst not stimulating differentiation.

**Fig 4:**
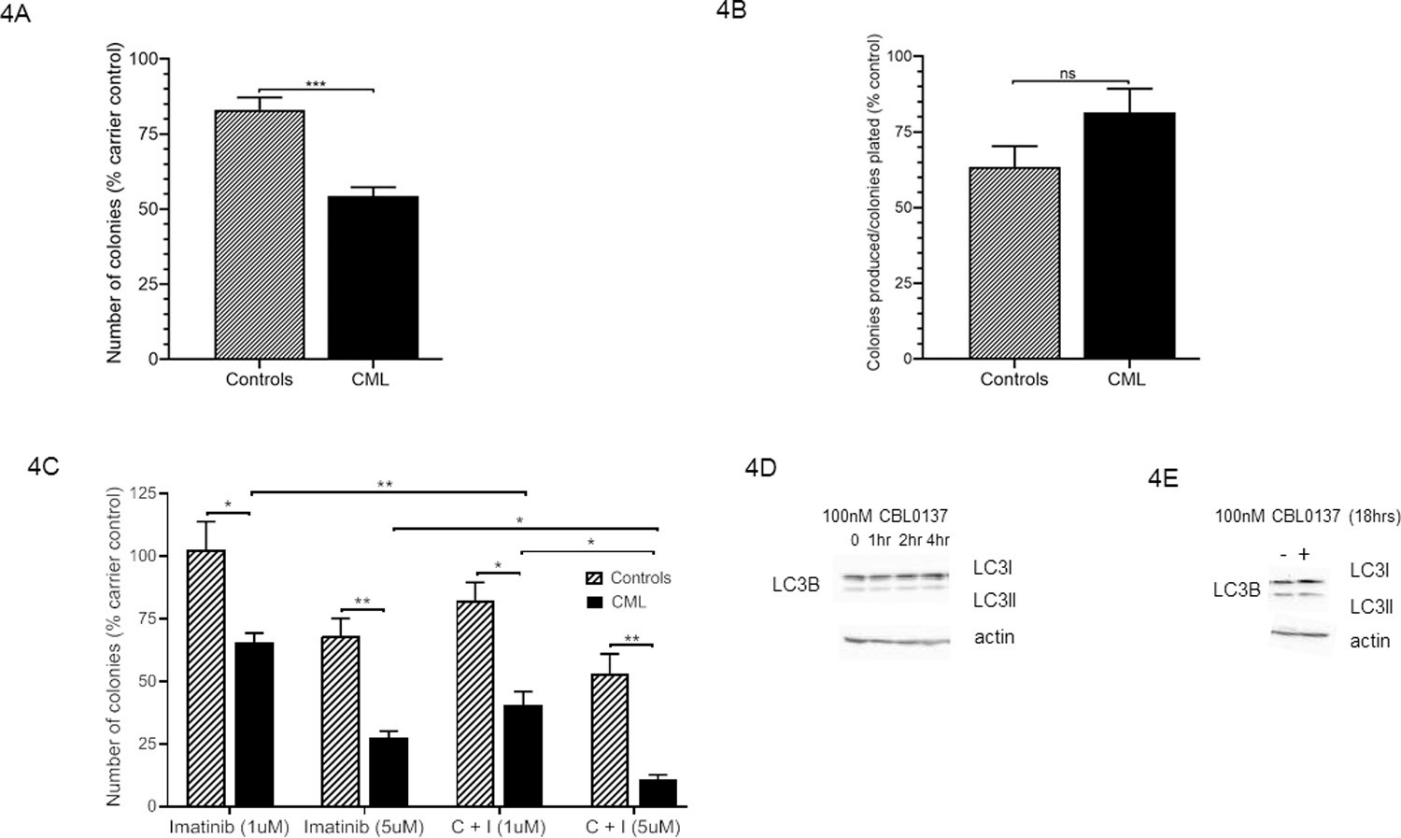
CBL0137 selectively reduces the clonogenic activity of CD34+ cells isolated from CML patients. Colony forming assays were performed in the presence or absence of 100nM CBL0137 with CD34^+^ cells enriched from control and CML patients in methylcellulose complete media (R&D systems) supplemented with 2u/ml EPO with cells at a density of 3000cells/ml (Fig 4A). Colonies were counted at day 14 and are expressed as a percentage of carrier control (mean±SEM, n = 6 for CML and n = 8 for healthy controls). The average number of colonies seen in carrier control was 71±14 for CML and 49±5 for healthy controls (mean±SEM). Colonies produced after 7 days were resuspended and replated in methylcellulose (Fig 4B). The number of colonies was assessed following 14 days and the data expressed as a percentage of control in relation to the number of colonies produced as a fraction of the colonies plated (mean±SEM, n = 6 for CML and n = 8 for healthy controls). The average number of colonies seen in carrier control was 47±6 for CML and 74±8 for healthy controls (mean±SEM). Colony forming assays were performed in the presence of 100nM CBL0137 with either 1μM or 5μM Imatinib with CD34^+^ cells enriched from control and CML patients (Fig 4C). Colonies were counted at day 14 and the results expressed as a percentage of carrier control (mean±SEM, n = 3). The average number of colonies seen in carrier control was 68±10 for CML and 75±12 for healthy controls (mean±SEM). The results of a t-test results are represented by; ns–not significant, *< 0.05, **<0.01, ***<0.001. Western blot analysis of LC3B expression in K562 cell lysates following either a four hour time course (Fig 4D) or 18 hour treatment (Fig 4E) with 100nM CBL0137.

Given the apparent differential effects on the primitive CD34 expressing cells we next investigated the ability of CBL0137 to preferentially inhibit haemopoietic colony formation in CML patient samples (Fig 4A). As we have demonstrated previously [18] CBL0137 has a minimal effect on colony formation in healthy controls at 100nM. It does however have a significant inhibitory effect on colony formation on cells from CML patients (Fig 4A) hence offering a potential novel therapy. The colonies produced were replated in the absence of any drug to see if the colonies not affected still contained primitive cells *ie* was there an effect on self-renewal (Fig 4B). No such effect was observed suggesting that the drug is inducing cell death not inducing stem cell commitment. This concurs with the data from the assays performed to quantify apoptosis (Fig 3C and 3D).

Given that TKIs rarely achieve therapy-free remission (as they fail to eradicate the leukemic stem cells) and issues with the development of drug resistance approaches other than targeting BCR/ABL have been proposed (reviewed in Massimino et al [26]). Furthermore it is envisaged that using drug combinations with TKIs will improve therapeutic outcome (reviewed in Westerweel et al [27] and several clinical trials are utilising such strategies eg Clinical Trial Identifier: NCT01657604, NCT02011945). Since TKIs are still the standard first line treatment for CML we further investigated the ability of CBL0137 to synergise with the TKI, Imatinib. CBL0137 (100nM) significantly improved the TKI-mediated inhibition of colony formation observed at 1μM and 5μM Imatinib (Fig 4C).

**Fig 5:**
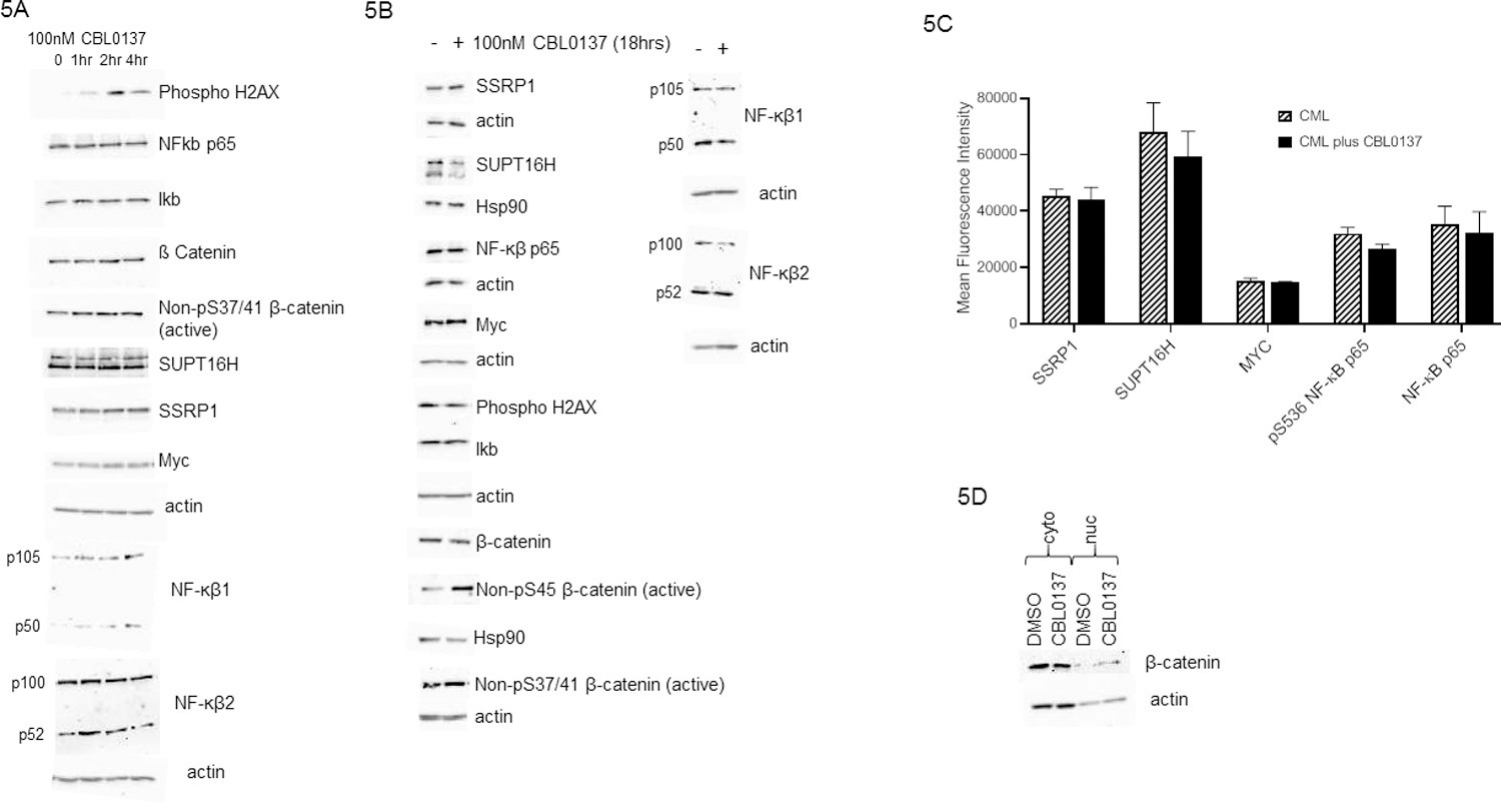
CBL0137 induces β-Catenin activation. Western blot analysis of the proteins indicated in K562 cell lysates following either a four hour time course (Fig 5A) or 18 hour (Fig 5B) treatment with 100nM CBL0137. Fig 5C; CD34^+^ cells isolated from CML patients were cultured in Fishers media supplemented with 5% Horse serum in the presence or absence of 100nM CBL0137 for 18 hours then stained with the antibodies to the proteins shown and expression levels analysed on a Novocyte flow cytometer (ACEA Biosciences) using FloJo software. Fig 5D; Western blotting analysis of β-catenin expression. K562 cells were treated with 100nM CBL0137 for 18 hours before nuclear and cytoplasmic proteins were enriched.

Having demonstrated the ability of CBL0137 to preferentially kill primitive CML cells we investigated its potential mechanism of action. Curaxins have been reported to target the FACT complex and act via p53, NF-kβ, MYC and β-catenin in other cancers [13–15]. However, whilst the use of curaxins for cancer treatment were first identified following a screen for non-genotoxic small molecules capable of restoring p53 activity [28] curaxin toxicity is not limited to cells expressing p53 [14]. One p53 independent mode of action for the curaxins reported in ovarian cancer is the activation of autophagy [29]. Since autophagy has been shown to protect CML stem cells following TKI treatment [30] and autophagy inhibitors shown to have promise as novel treatments for elimination of persistent CML stem cells [31] we were keen to dismiss any potential autophagy activation by curaxins in the context of CML. Using LC3B expression as a marker of autophagy activation we failed to observe any changes in expression/activity over either a four (Fig 4D) or 18 hour period (Fig 4E). Having ruled out the activation of autophagy as the mode of action of curaxin in CML we undertook analysis on the expression of the components of the FACT complex and other reported targets of curaxin. These targets were investigated following treatment with CBL0137 in K562 cells over a four hour (Fig 5A) or 18 hour time course (Fig 5B). No effect was seen on the expression levels of the subunits of the FACT complex SSRP1 and SUPT16H which fits with a proposed mechanism of action for curaxins which is the chromatin trapping of the FACT complex [14]. More interestingly perhaps no effect was seen on either NF-ĸB signalling or Myc expression (Fig 5B). This lack of effect of CBL0137 on the expression of these pathways was recapitulated in primary CD34+ cells isolated from patients with CML (Fig 5C). Whilst curaxins are routinely quoted as acting via NF-ĸB inhibition it was originally only shown to have such an effect via reporter assays [14]. Curaxins may therefore inhibit the transcriptional activity of NF-ĸB via direct interference with its transcriptional or post-transcriptional activity and not activation/translocation. We can therefore not completely rule out a role for NF-ĸB inhibition in the effects we observe. Whilst failing to see perturbation of the major reported targets of curaxin we did see an increase in phospho-Histone H2A.X (ser139) indicative of DNA damage. DNA damage was detectable two hours post curaxin treatment (Fig 5A) but had reduced to pre-treatment levels after 18 hours (Fig 5B). Whilst curaxins were initially reported not to cause DNA damage [14] and this was not observed in neuroblastoma cells [13] more recently CBL0137 has been shown to cause DNA damage in Glioblastoma [32]. This is perhaps not surprising given the role that the FACT complex plays in the DNA damage response to both double and single strand breaks [33,34]. In their work on Glioblastoma cells Tallman et al [32] propose that the curaxin induced chromatin trapping [14] of the FACT complex reduces the pool of FACT available to respond to DNA damage. This may be occurring in the hematopoietic cells we are studying.

An effect we observed following 18 hours CBL0137 treatment (Fig 5B) was a decrease in the phosphorylation of β-catenin at serine 45 which is indicative of β-catenin activation. However, we failed to detect any change in phosphorylation of β-catenin at Ser 37/41 (Fig 5A and 5B). These two phosphorylation sites control the degradation of β-catenin [35–37]. Phosphorylation at Serine 45 by CK1 primes β-catenin for subsequent phosphorylation of Serine 37 and Threonine 41 which destabilises β-catenin. The lack of change in β-catenin phosphorylation at ser37/41 is matched by the lack of change in the overall level of β-catenin (Fig 5A and 5B).

To further investigate the effects of CBL0137 on β-catenin activation we enriched nuclear and cytoplasmic fractions from K562 cells treated with CBL0137 and undertook western blot analysis. CBL0137 treatment leads to a nuclear translocation of β-catenin (Fig 5D) suggesting that its differential effects on CML may be via β-catenin signalling. Given the potential role of β-catenin in TKI resistance in CML further work is required to understand the consequences of this curaxin induced β-catenin activation. The role of β-catenin in CML is complicated and it has been shown that whilst β-catenin inactivation synergizes with imatinib to delay disease recurrence it does not increase survival after CML initiation in murine models [16].

Our preliminary observations demonstrating the enhanced inhibition of CML colony formation, induction of DNA damage and interference of β-catenin signalling highlight the potential utility of CBL0137, a well-tolerated drug, for the treatment of CML either alone or in combination with TKIs. Given the fact that curaxins are already proving their worth in the treatment of other malignancies and the proposal that a combination of β-catenin inhibitors and TKIs may be useful to eradicate leukaemic stem cells our study provides evidence to support the initiation of further studies on the use of curaxin in CML treatment.

## Supporting information

S1 Fig mRNA expression of SSRP1 in quiescent cells. Data extracted from Affer et al (*J Oncol*. 2011;2011:798592–798592). Affer et al employed Hoechst 33342 and Pyronin Y to enrich CD34+ cells in GO from patients in chronic phase CML and healthy controls. RNA measurements were made utilising Affymetrix Human Genome U133 plus 2.0 arrays. Results are displayed as mean+/-SEM of log_2_ expression intensity (n = 5 for CML and n = 4 for normal patients). SSRP1 shows on average a 3.4 fold increase in expression at the mRNA level in CML quiescent cells compared to quiescent cells isolated from healthy controls. The results of a t-test are shown **<0.01.
(DOCX)

S2 Fig Original full images for western blots. Complete images for the western blots shown in Fig 5. Following incubation with the appropriate HRP-linked secondary antibodies and Supersignal West Pico Plus chemiluminescent substrate (ThermoScientific) protein expression was visualised on a Chemidoc XRS (BioRad) using Quantity One software.
(PDF)
