# Supplementary material for: Combination of curaxin and tyrosine kinase inhibitors display enhanced killing of primitive Chronic Myeloid Leukaemia cells
Source: PLoS One. 2022 Mar 31;17(3):e0266298. doi: 10.1371/journal.pone.0266298 (PMC8970494; doi:10.1371/journal.pone.0266298)
Supplement: S1 Fig — Data extracted from Affer et al (J Oncol. 2011;2011:798592–798592). Affer et al employed Hoechst 33342 and Pyronin Y to enrich CD34+ cells in GO from patients in chronic phase CML and healthy controls. RNA measurements were made utilising Affymetrix Human Genome U133 plus 2.0 arrays. Results are displayed as mean+/-SEM of log2 expression intensity (n = 5 for CML and n = 4 for normal patients). SSRP1 shows on average a 3.4 fold increase in expression at the mRNA level in CML quiescent cells compared to quiescent cells isolated from healthy controls. The results of a t-test are shown **<0.01. (DOCX) [file pone.0266298.s001.docx]

**SUPPLEMENTARY INFORMATION**

**Supplementary Figure 1: mRNA expression of SSRP1 in quiescent cells**

Data extracted from Affer et al (*J Oncol.* 2011;2011:798592-798592). Affer et al employed Hoechst 33342 and Pyronin Y to enrich CD34+ cells in GO from patients in chronic phase CML and healthy controls. RNA measurements were made utilising Affymetrix Human Genome U133 plus 2.0 arrays. Results are displayed as mean+/-SEM of log_2_ expression intensity (n=5 for CML and n=4 for normal patients). SSRP1 shows on average a 3.4 fold increase in expression at the mRNA level in CML quiescent cells compared to quiescent cells isolated from healthy controls. The results of a t-test are shown **<0.01.
