# Supplementary material for: Combination of curaxin and tyrosine kinase inhibitors display enhanced killing of primitive Chronic Myeloid Leukaemia cells
Source: PLoS One. 2022 Mar 31;17(3):e0266298. doi: 10.1371/journal.pone.0266298 (PMC8970494; doi:10.1371/journal.pone.0266298)
Supplement: S2 Fig — Complete images for the western blots shown in Fig 5. Following incubation with the appropriate HRP-linked secondary antibodies and Supersignal West Pico Plus chemiluminescent substrate (ThermoScientific) protein expression was visualised on a Chemidoc XRS (BioRad) using Quantity One software. (PDF) [file pone.0266298.s002.pdf]

S2 Fig. Original full images for western blots.

Complete images for the western blots shown in Fig 4 and 5. Following incubation with the appropriate HRP-linked secondary antibodies and Supersignal West Pico Plus chemiluminescent substrate (ThermoScientific) protein expression was visualised on a Chemidoc XRS (BioRad) using Quantity One software

Western blots in Fig 4D

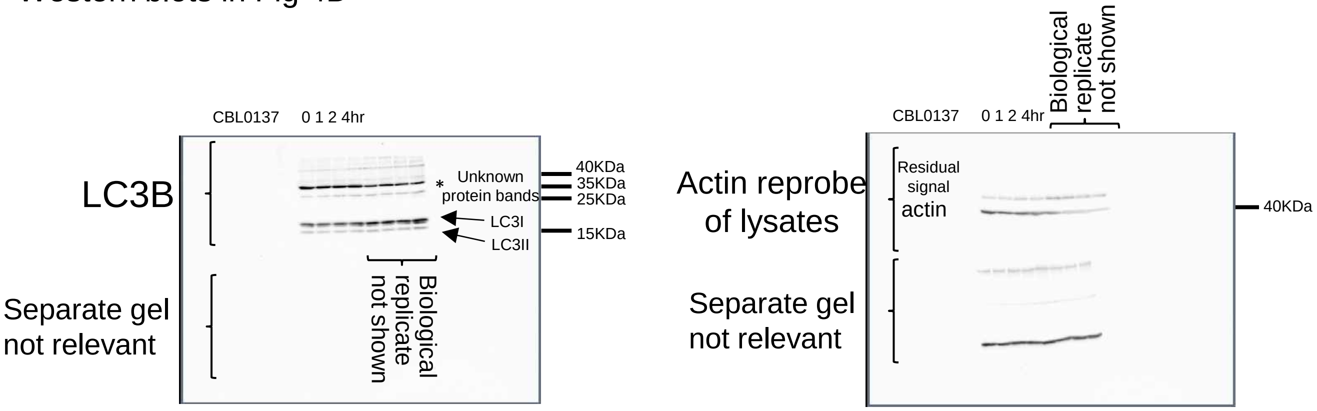

Western blots in Fig 4E

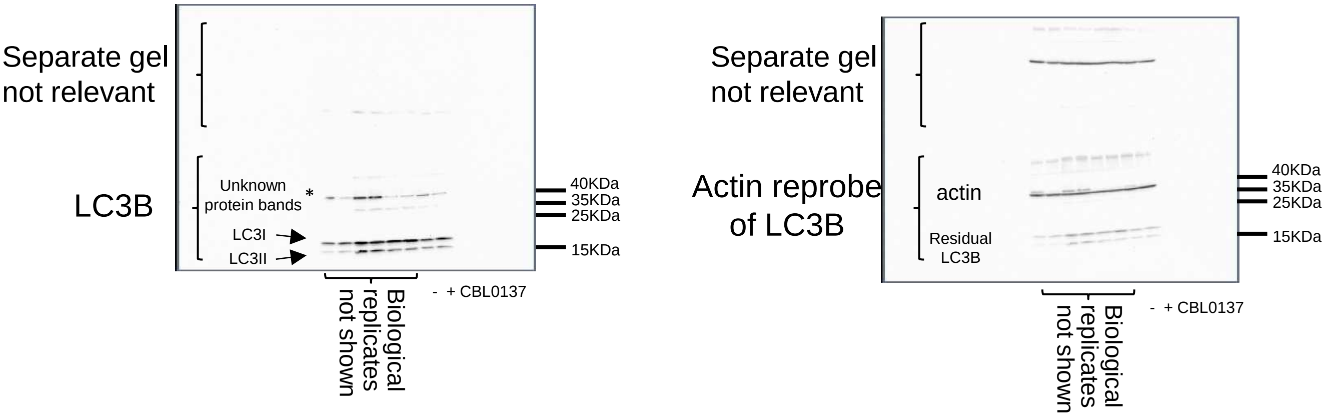

Western blots in Fig 5A

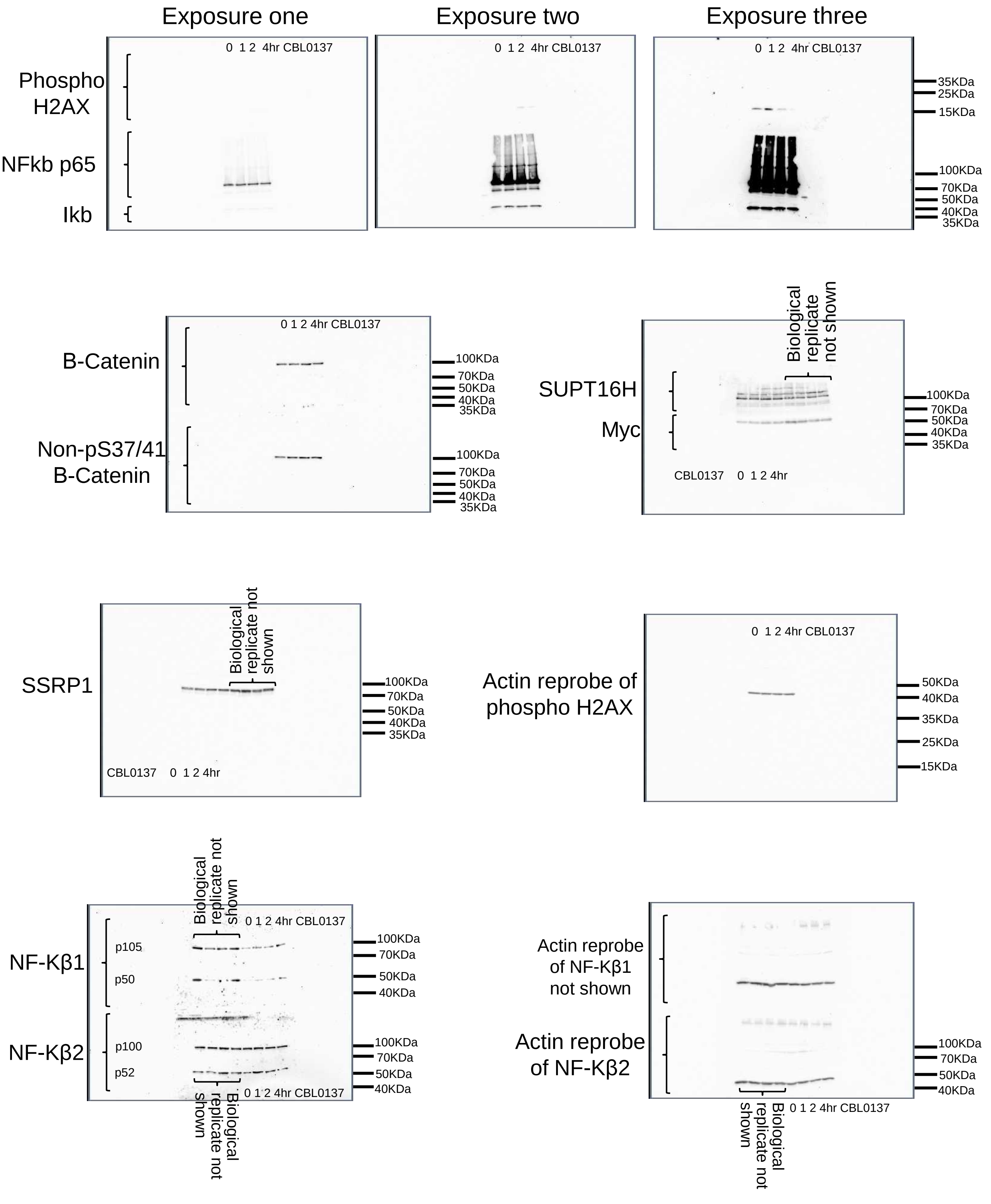

Western blots in Fig 5B

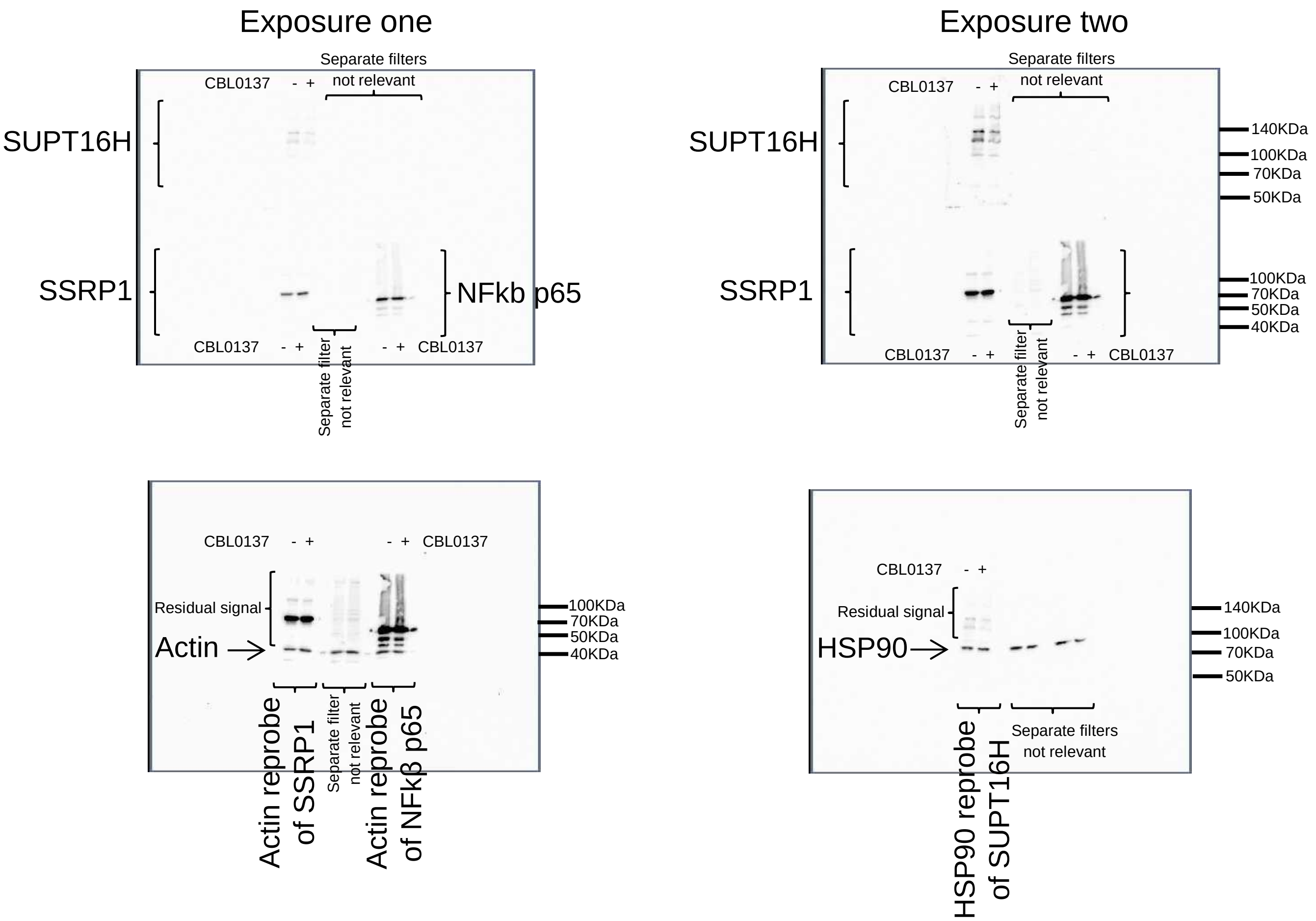

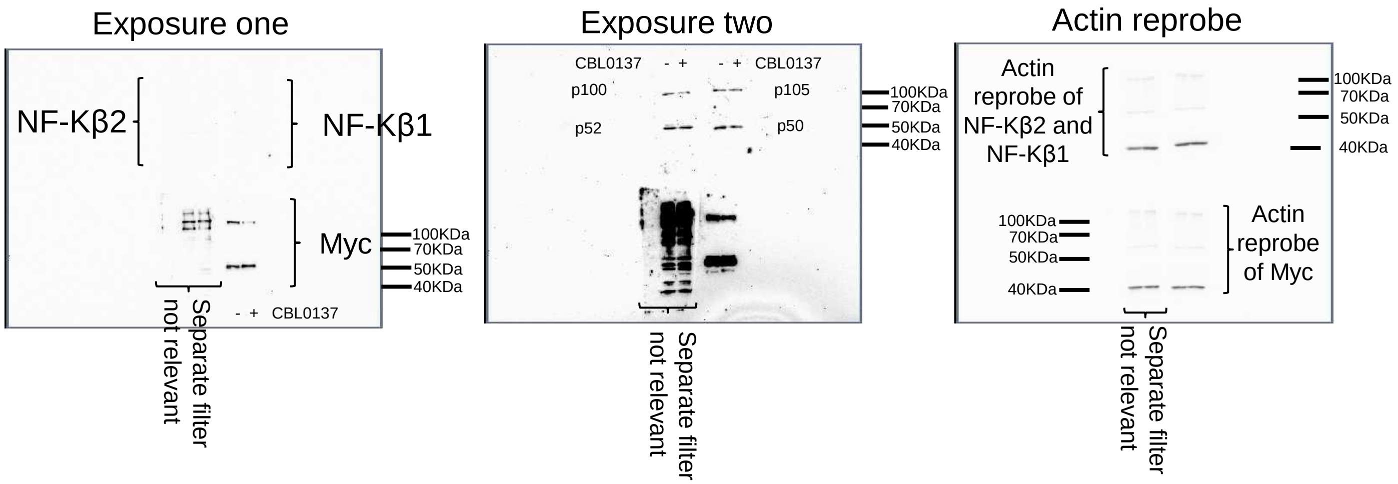

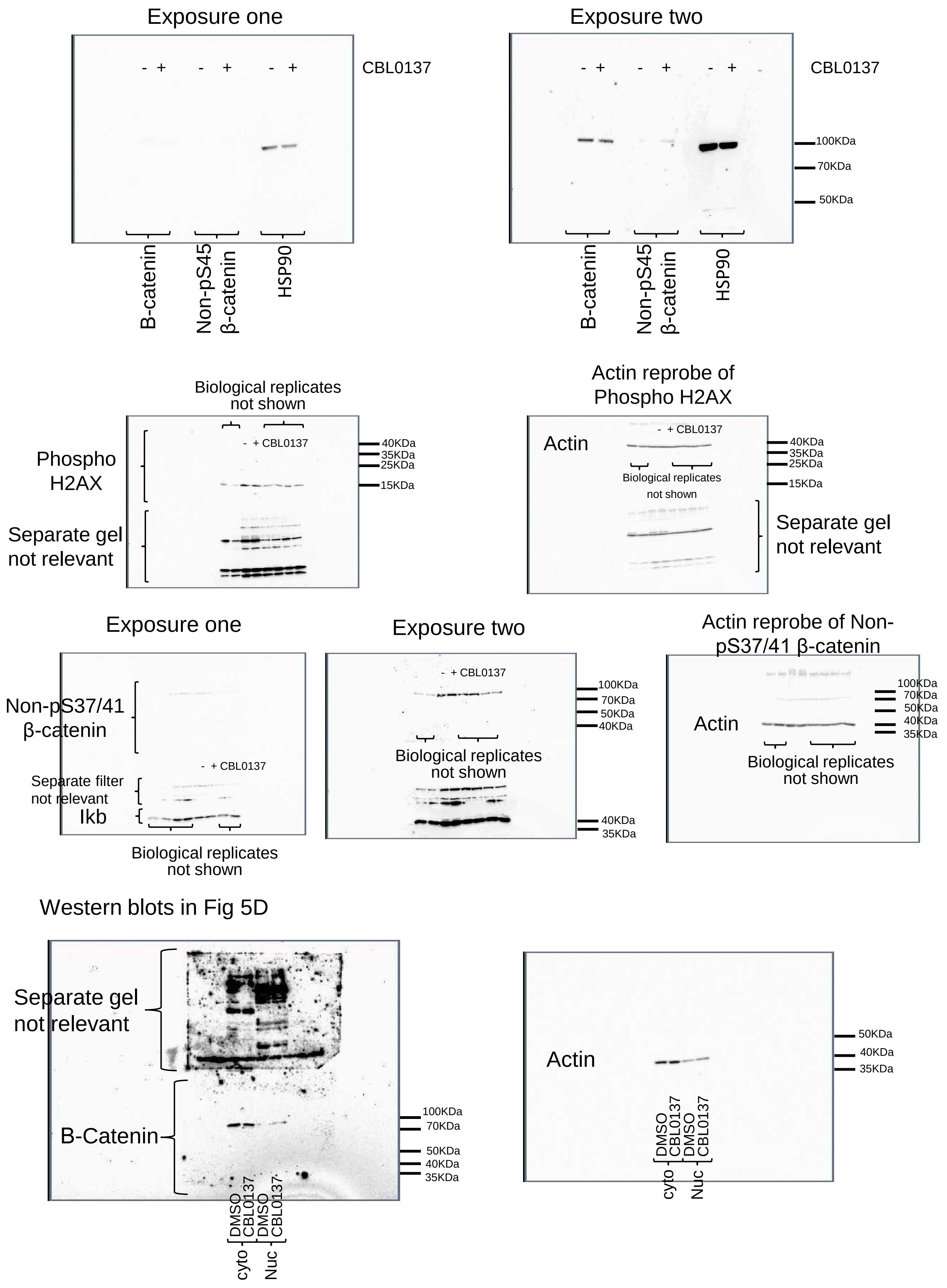
Exposure one
- + - + - + CBL0137
B-catenin
Non-pS45 β-catenin
HSP90
Exposure two
- + - + - + CBL0137
100KDa
70KDa
50KDa
B-catenin
Non-pS45 β-catenin
HSP90
Biological replicates not shown
Phospho H2AX
- + CBL0137
40KDa
35KDa
25KDa
15KDa
Separate gel not relevant
Actin reprobe of Phospho H2AX
Actin
- + CBL0137
40KDa
35KDa
25KDa
15KDa
Biological replicates not shown
Separate gel not relevant
Exposure one
Non-pS37/41 β-catenin
- + CBL0137
Separate filter not relevant
Ikb
Biological replicates not shown
Exposure two
- + CBL0137
100KDa
70KDa
50KDa
40KDa
Biological replicates not shown
40KDa
35KDa
Actin reprobe of Non-pS37/41 β-catenin
Actin
100KDa
70KDa
50KDa
40KDa
35KDa
Biological replicates not shown
Western blots in Fig 5D
Separate gel not relevant
B-Catenin
100KDa
70KDa
50KDa
40KDa
35KDa
DMSO
CBL0137
DMSO
CBL0137
cyto
Nuc
Actin
50KDa
40KDa
35KDa
DMSO
CBL0137
DMSO
CBL0137
cyto
Nuc
